# Carbonic Anhydrase Inhibitors. Part 55^1^ Metal Complexes of 1,3,4-Thiadiazole-2-Sulfonamide Derivatives: *In Vitro* Inhibition Studies With Carbonic Anhydrase Isozymes I, II and IV

**DOI:** 10.1155/MBD.1998.103

**Published:** 1998

**Authors:** Claudiu T. Supuran, Andrea Scozzafava, Fabrizio Briganti, Marc A. Ilies, Andrei Jitianu

**Affiliations:** 1 Università degli Studi Dipartimento di Chimica Laboratorio di Chimica Inorganica e Bioinorganica Via Gino Capponi 7 Florence I-50121 Italy; 2 University of Bucharest Department of Chemistry Bd. Republicii 13 Bucharest Romania; 3 “I.G. Murgulescu” Institute of Physical Chemistry Academia Romana Spl. Independentei 202 Bucharest R-77208 Romania

## Abstract

Coordination compounds of 5-chloroacetamido-1,3,4-thiadiazole-2-sulfonamide (Hcaz) with V(IV), Cr(lll), Fe(ll), Co(ll), Ni(ll) and Cu(ll) have been prepared and characterized by standard procedures (spectroscopic, magnetic, EPR, thermogravimetric and conductimetric measurements). Some of these compounds showed very good *in vitro* inhibitory properties against three physiologically relevant carbonic anhydrase (CA)isozymes, i.e., CA I, II, and IV. The differences between these isozymes in susceptibility to inhibition by these metal complexes is discussed in relationship to the characteristic features of their active sites, and is rationalized in terms useful for developing isozyme-specific CA inhibitors.


### CARBONIC ANHYDRASE INHIBITORS. Part 55[1]
### METAL COMPLEXES OF 1,3,4-THIADIAZOLE-2-SULFONAMIDE DERIVATIVES: *IN VITRO* INHIBITION STUDIES WITH CARBONIC ANHYDRASE ISOZYMES I, II and IV

Claudiu T. Supuran[1]*, Andrea Scozzafava[1], Fabrizio Briganti[1], Marc A. Ilies[2] and Andrei Jitianu[3]

[1] Università degli Studi, Dipartimento di Chimica, Laboratorio di Chimica Inorganica e Bioinorganica, Via Gino Capponi 7, I-50121, Florence, Italy
[2] University of Bucharest, Department of Chemistry, Bd. Republicii 13, Bucharest, Roumania
[3] "I.G. Murgulescu" Institute of Physical Chemistry, Academia Romana, Spl. Independentei 202, R-77208 Bucharest, Roumania.

**Abstract**: Coordination compounds of 5-chloroacetamido-1,3,4-thiadiazole-2-sulfonamide (Hcaz) with V(IV), Cr(III), Fe(II), Co(II), Ni(II) and Cu(II) have been prepared and characterized by standard procedures (spectroscopic, magnetic, EPR, thermogravimetric and conductimetric measurements). Some of these compounds showed very good *in vitro* inhibitory properties against three physiologically relevant carbonic anhydrase (CA) isozymes, i.e., CA I, II, and IV. The differences between these isozymes in susceptibility to inhibition by these metal complexes is discussed in relationship to the characteristic features of their active sites, and is rationalized in terms useful for developing isozyme-specific CA inhibitors.

### Introduction

1,3,4-Thiadiazole-2-sulfonamide derivatives[2,3] played a critical role for the development of several important classes of pharmacological agents, such as the diuretics with saluretic action,[4] benzothiadiazine and high-ceiling diuretics,[4-6] and the antiglaucoma drugs with carbonic anhydrase (CA) inhibitory action among others.[7-9] The prototype of all these drugs was constituted by acetazolamide **1**, the first non-mercurial diuretic,[4] used for more than 45 years in clinical medicine as diuretic, antiglaucoma,[7] antiepileptic[10] and antiulcer compound.[11] It is still used nowadays, mainly as a diagnostic tool in NMR imaging,[12] and in many physiological studies.[13]

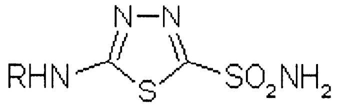

**1-4**

**1**: R = Ac

**2**: R = $PhSO_2$

**3**: R = $ClCH_2CO$

**4**: R = adamantyl-1-CO

The success of acetazolamide in clinical medicine fostered much research in the synthesis and pharmacological evaluation of many 1,3,4-thiadiazole-2-sulfonamide derivatives.[14-16] Thus, benzolamide **2** was approved in USA as a drug, and several studies showed its efficiency in emphysema,[17] but its status is nowadays that of an orphan drug, although its clinical value has never been contested. Benzolamide is also widely used in many renal and pulmonary physiological studies.[13,18] The halogeno-acetazolamides, such as chloroacetazolamide **3** led to the development of topical antiglaucoma sulfonamide CA inhibitors,[13a] which recently introduced in clinics, constituted one of the best treatments of open-angle glaucoma.[19,20]

Metal complexes of sulfonamides of type **1-3** started to be investigated only in the last 10 years, with the report of Borras' group of the first X-ray crystallographic structure of some acetazolamide complexes.[21] It has been thereafter proved by our group[22-25] that such compounds possess unexpectedly strong inhibitory properties against the zinc enzyme carbonic anhydrase (CA, EC 4.2.1.1), and this finding opened new vistas in the search of isozyme-specific inhibitors, as well as other types of clinical/diagnostic applications of such enzyme inhibitors.[16,26]

Thus, we have recently reported the potent intraocular pressure (IOP) lowering effects of metal complexes of sulfonamide CA inhibitors such as chloracetazolamide **3**,[1] the adamantyl-carboxamido compound **4**,[26] as well as other classes of heterocyclic sulfonamides.[27] Metal ions that generally led to potent such activities were Zn(II) and Cu(II).[1,26,27] The most remarkable aspect of these studies was that even when the sulfonamide from which the metal complexes were prepared was ineffective topically as an IOP lowering agent, its metal complexes possessed powerful such properties.[1,26,27] It has been proved that the observed effects are due to inhibition of at least two CA isozymes (CA II and IV) present within ciliary processes of the eye in the experimental animals used.[26] Thus, these types of compounds constitute interesting candidates for developing novel antiglaucoma drugs.[26]

Since many CA isozymes are presently known,[28-30] and not all of them were tested for interaction with the metal complexes of heterocyclic sulfonamides, it appeared of interest to study in more details this phenomenon which might be critical for the design of more effective and specific, eventually isozyme-specific CA inhibitors. In this paper we report the preparation of metal complexes of the conjugate base of sulfonamide **3**, containing the following metal ions: V(IV), Cr(III), Fe(II), Co(II), Ni(II) and Cu(II). The obtained complexes have been characterized by standard procedures (spectroscopic, EPR, magnetic, thermogravimetric and conductimetric measurements) and assayed for inhibition of three CA isozymes, i.e., CA I, II, and IV.

### Materials and Methods

IR spectra were recorded on a Perkin-Elmer 16PC FTIR instrument, in the range 200-4000 $cm^{-1}$, in KBr pellets. Solution electronic spectra were recorded with a Cary 3 spectrophotometer interfaced with a PC. Electronic spectra were obtained by the diffuse reflectance technique in MgO as reference, with a Perkin Elmer Lambda 15 apparatus, in the range 200-900 $cm^{-1}$. Conductimetric measurements were done in DMF solutions, at 25°C (concentrations of 1 mM of complex) with a Fisher conductimeter. EPR spectra were recorded on a Varian E-9 spectrometer at room temperature, in crystalline powder. The field was calibrated using crystalline diphenylpicrylhydrazyl (g = 2.0036). Magnetic susceptibility measurements were carried out at room temperature with a fully automated AZTEC DSM8 pendulum-type susceptometer. Mercury(II) tetrakis-(thiocyanato)cobaltate(II) was used as a susceptibility standard. Corrections for the diamagnetism were estimated from Pascal's constants.[31] Elemental analyses were done by combustion for C,H,N with an automated Carlo Erba analyzer, and gravimetrically for the metal ions, and were ± 0.4% of the theoretical values. Thermogravimetric measurements were done in air, at a heating rate of 10°C/min., with a Perkin Elmer 3600 thermobalance.

Chloracetazolamide was prepared as described in a previous paper,[1] whereas other sulfonamides used as standards in the enzymatic assays were commercially available from Aldrich, Sigma or Acros. Metal salts (iron(III) chloride dihydrate; vanadyl(IV) sulfate dihydrate; cobalt(II) chloride hexahydrate; cobalt(II) acetate; nickel(II) chloride; chromium(III) chloride hexahydrate and copper(II) chloride and acetate dihydrate) were from E. Merck.

Human CA I and CA II cDNAs were expressed in *Escherichia coli* strain BL21 (DE3) from the plasmids pACA/HCA I and pACA/HCA II (the two plasmids were a gift from Prof. Sven Lindskog, Umea University, Sweden). Cell growth conditions were those described by Lindskog's group,[32] and enzymes were purified by affinity chromatography according to the method of Khalifah et al.[33] Enzyme concentrations were determined spectrophotometrically at 280 nm, using a molar absorptivity of 49 $mM^{-1}$. $cm^{-1}$ for CA I and 54 $mM^{-1}.cm^{-1}$ for CA II, respectively, based on $M_r$ = 28.85 kDa for CA I, and 29.3 kDa for CA II, respectively.[34] CA IV was isolated from bovine lung microsomes.[35]

Initial rates of 4-nitrophenyl acetate hydrolysis were monitored spectrophotometrically, at 400 nm and 25°C, with a Cary 3 apparatus interfaced with an IBM compatible PC by the method of Pocker and Stone.[36]. Solutions of substrate were prepared in anhydrous acetonitrile; the substrate concentrations varied between $10^{-2}$ and $10^{-6}$ M. A molar absorption coefficient $\varepsilon$ = 18,400 $M^{-1}.cm^{-1}$ was used for the 4-nitrophenolate formed by hydrolysis, in the conditions of the experiments (pH 7.80), as reported by Pocker and Stone.[36] Non-enzymatic hydrolysis rates were always subtracted from the observed rates. Duplicate experiments were done for each inhibitor, and the values reported throughout the paper are the averages of such results. $IC_{50}$ represents the molarity of inhibitor producing a 50% decrease of enzyme catalyzed hydrolysis of 4-nitrophenyl acetate.

### General procedure for the preparation of complexes 5 - 17

An amount of 10 mM of sulfonamide **3** was dissolved in 50 mL of methanol-water (1:1, v/v). The stoichiometric amount of metal salt dissolved in a small amount (5 mL) of water was added (a molar ratio sulfonamide:metal salt of 1:1; 2:1 and 3:1 has been used in different experiments), and the mixture was treated with the stoichiometric amount of KOH (in order to obtain the monopotassium salt of the original sulfonamide) or with an excess of 25% acqueous amonia. The reaction mixture has been heated on a steam bath for 1-3 hours, then left overnight and the obtained precipitates were filtered and air dried. An exception from the above procedure was the preparation of complex **9**, case in which *trans*-$[Co(en)_2Cl_2]Cl$ has been used instead of a simple cobalt salt.

### Results and Discussion

Complexes **5-17** prepared in the present study are shown in Table I, together with their elemental analysis data.

Table I: Prepared complexes **5-17**, containing the conjugate bases of sulfonamide **3** as ligand and their elemental analysis data. L stands for the sulfonamide deprotonated species of **3**, whereas tda for the sulfonamide deprotonated species of 5-amino-1,3,4-thiadiazole-2-sulfonamide, the hydrolysis product of **3**.

| No. | Complex | Analysis (calculated/found) | | | |
|---|---|---|---|---|---|
| | | %M[a] | %C[b] | %H[b] | %N[b] |
| **5** | $K_2[VOL_4]$ | 4.3/4.5 | 16.4/16.1 | 1.3/1.3 | 19.1/19.0 |
| **6** | $[Fe(HL)_2Cl_2]$ | 8.7/8.5 | 15.0/14.8 | 1.5/1.6 | 17.5/17.5 |
| **7** | $[CoL_2(NH_3)_2]$ | 9.7/10.0 | 15.8/15.4 | 2.3/2.2 | 23.1/23.0 |
| **8** | $[Co_2L_2(OH_2)_2](OH)_2$ | 16.8/17.1 | 13.7/13.6 | 2.0/2.2 | 16.0/15.8 |
| **9** | $[CoL_2Cl_2(OH_2)_2]$ | 8.7/8.7 | 14.1/14.1 | 1.7/1.6 | 16.5/16.4 |
| **10** | $[CoL_4(OH_2)_2]$ | 5.2/5.4 | 17.1/16.9 | 1.7/1.6 | 20.0/19.7 |
| **11** | $[NiL_2(NH_3)_2]$ | 9.7/9.4 | 15.8/15.8 | 2.3/2.6 | 23.1/23.0 |
| **12** | $[NiL_2(OH_2)_2]$ | 9.6/9.6 | 15.7/15.8 | 1.9/1.5 | 18.4/18.3 |
| **13** | $[Cu_2(HL)_2Cl_4]$ | 20.9/20.5 | 12.2/11.9 | 1.2/1.3 | 14.3/14.1 |
| **14** | $[Cu_2L(AcO)_2]$ | 25.3/25.5 | 19.1/19.2 | 2.0/1.8 | 11.1/11.2 |
| **15** | $[Cu_2L(AcO)_2]_n$ | 25.3/25.1 | 19.1/18.9 | 2.0/2.1 | 11.1/10.8 |
| **16** | $[Cr_2(Htda)_2Cl_4(OH)_2]$ | 16.2/16.2 | 7.5/7.5 | 1.5/1.3 | 17.4/17.5 |
| **17** | $[Cr(Htda)_2Cl_2]Cl \cdot 8\,H_2O$ | 7.8/7.9 | 7.2/7.1 | 3.6/4.0 | 16.8/16.5 |

[a]By gravimetry; [b]By combustion.

From the above data one can note several facts. Thus, although the synthesis have been started from Fe(III) and Co(III) salts (for preparing compounds **6** and **8, 9**) the obtained derivatives contained Fe(II) and Co(II) ions, respectively, due to reduction of the metal ions in the conditions used in synthesis. This side reaction was anyhow of help, since Fe(II) complexes of heterocyclic sulfonamides have not been studied previously, in contrast to Fe(III) complexes which were much investigated.[22-24] Synthesis in which ammonia has been used as deprotonating agent, led to the formation of metal complexes in which this molecule was present in the coordination sphere, in addition to the sulfonamide anion(s). The anion of the metal salt from which the complexes were prepared was also of much importance in the synthesis, especially for the Co(II) and Cu(II) derivatives, cases in which complexes with a large variety of compositions could be prepared by slight variations of the synthetic procedure described above and the metal salt employed in the preparations. In the case of the Cr(III) derivatives, a hydrolysis of the original ligand was evidenced, with formation of 5-amino-1,3,4-thiadiazole-2-sulfonamide (Htda) which subsequently complexed (as neutral ligand) this metal ion. Such a behaviour was previously evidenced for the complexation of divalent metal ions (such as Mg(II) and Mn(II)) by compound **3**.[1]

Electronic spectroscopic (by the diffuse reflectance technique) data for complexes **6-16** are shown in Tables II - IV, together with the assignements of the observed transitions. For the V(IV) complex **5** the typical transitions of V(IV) in square pyramidal geometry were evidenced in the electronic spectrum,[37] at 26,315; 15,480 and 11,590 $cm^{-1}$, respectively, whereas for the two Cr(III)

derivatives **16** and **17**, the electronic spectral transitions are shown in Table IV, proving the presence of octahedral chromium ions in these derivatives.[38]

The electronic spectroscopic data for the Fe(II), Co(II) and Ni(II) derivatives (Table II), although generally more complicated than for the systems mentioned above, permitted to evidence the presence of octahedral metal ions in many of the prepared complexes (such as **6**, **7**, **9-12**), whereas for some Co(II) derivatives (such as **8-10**), tetrahedral geometries were also found.[39,40] Proposed geometries for some of these metal ions in the prepared complexes are shown schematically below.

Table II: Electronic spectroscopic data for compounds **6-12**.

| Compound (colour) | Abs. max. λ (nm) | Wavenumber ν ($cm^{-1}$) | Assignments |
|---|---|---|---|
| **6** | 380 | 26,315 | $\pi\rightarrow\pi^*$ + CT# |
| (yellow) | 501 | 20,000 | $^1A_{1g}\rightarrow{}^1T_{1g}$ ($D_{4h}$) |
| | 663 | 15,100 | $^1A_{1g}\rightarrow{}^1A_{2g}$ ($D_{4h}$) |
| | 1055 | 9,478 | $^1A_{1g}\rightarrow{}^1E_g$ ($D_{4h}$) |
| **7** | 380 | 26,315 | $\pi\rightarrow\pi^*$ + CT (M→L) |
| (pink) | 531 | 18,840 | $^4T_{1g}\rightarrow{}^4T_{1g}$ (P) ($O_h$) |
| | 1111 | 9,000 | $^4T_{1g}\rightarrow{}^4T_{2g}$ ($O_h$) |
| **8** | 380 | 26,315 | $\pi\rightarrow\pi^*$ + CT (M→L) |
| (black) | 490 | 20,410 | $^4T_{1g}\rightarrow{}^4T_{1g}$ (P) ($O_h$) |
| | 608 | 16,450 | $^4A_2\rightarrow{}^4T_1$ (F) ($T_d$) |
| | 1181 | 8,470 | [$^4T_{1g}\rightarrow{}^4T_{2g}$ (P) ($O_h$)]+ [$^4A_2\rightarrow{}^4T_1$ (F) ($T_d$)] |
| **9** | 380 | 26,315 | $\pi\rightarrow\pi^*$ + CT (M→L) |
| (gray) | 480 | 20,830 | $^4T_{1g}\rightarrow{}^4T_{1g}$ (P) ($O_h$) |
| | 660 | 15,150 | $^4A_2\rightarrow{}^4T_1$ (P) ($T_d$) |
| | 1250 | 8,000 | [$^4T_{1g}\rightarrow{}^4T_{2g}$ (P) ($O_h$)]+ [$^4A_2\rightarrow{}^4T_1$ (T) ($T_d$)] |
| **10** | 380 | 26,315 | $\pi\rightarrow\pi^*$ + CT (M→L) |
| (pink) | 480 | 20,830 | $^4T_{1g}\rightarrow{}^4T_{1g}$ (P) ($O_h$) |
| | 660 | 15,150 | $^4A_2\rightarrow{}^4T_1$ (P) ($T_d$) |
| | 1250 | 8,000 | [$^4T_{1g}\rightarrow{}^4T_{2g}$ (P) ($O_h$)]+ [$^4A_2\rightarrow{}^4T_1$ (F) ($T_d$)] |
| **11** | 380 | 26,315 | ($\pi\rightarrow\pi^*$) +[$^3A_{1g}\rightarrow{}^3T_{1g}$ (P)] |
| (green) | 428 | 23,364 | $\pi\rightarrow\pi^*$ |
| | 638 | 15,673 | $^3A_{2g}\rightarrow{}^3T_{1g}$ |
| | 819 | 12,210 | $^3A_{2g}\rightarrow{}^1E_g$ |
| | 1100 | 9,090 | $^3A_{2g}\rightarrow{}^3T_{2g}$ |
| **12** | 380 | 26,315 | ($\pi\rightarrow\pi^*$) +[$^3A_{1g}\rightarrow{}^3T_{1g}$ (P)] |
| (green) | 625 | 16,000 | $^3A_{2g}\rightarrow{}^3T_{1g}$ |
| | 740 | 19,513 | $^3A_{2g}\rightarrow{}^1E_g$ |
| | 1104 | 9,057 | $^3A_{2g}\rightarrow{}^3T_{2g}$ |

### CT = charge transfer band.

Table III: Electronic spectroscopic and room temperature magnetic data for complexes **13-15**.

| Complex (colour) | $\mu_{eff}$ (BM) | Wavenumber $\nu$ (cm$^{-1}$) | Assignments |
|---|---|---|---|
| **13** (green) | 2.44 | 26,315<br>14,672<br>11,215 | $\pi \rightarrow \pi^*$ + CT[#]<br>$d_{yz} \rightarrow d_z$<br>$d_{yz} \rightarrow d_{xy}$ |
| **14** (grayish-green) | 1.43 | 29,037<br>20,355<br>15,163<br>10,000 | $\pi \rightarrow \pi^*$ + CT + $d_z \rightarrow d_{x-y}$<br>$\pi \rightarrow \pi^*$<br>$d_z \rightarrow d_{yz}$<br>$d_z \rightarrow d_{xy}$ |
| **15** (dark green) | 1.62 | 26,315<br>15,043<br>11,519 | $\pi \rightarrow \pi^*$ + CT + $d_z \rightarrow d_{x-y}$<br>$d_z \rightarrow d_{yz}$<br>$d_z \rightarrow d_{xy}$ |

[#] CT = charge transfer band.

Table IV: Electronic spectroscopic data for the Cr(III) complexes **16** and **17**.

| Complex (colour) | Abs. max. $\lambda$ (nm) | Wavenumber $\nu$ (cm$^{-1}$) | Assignment |
|---|---|---|---|
| **16** (dark green) | 418<br>567<br>667 | 23,930<br>17,640<br>14,995 | $^4A_{2g} \rightarrow {}^4T_{1g}$<br>$^4A_{2g} \rightarrow B_{2g}$ ($D_{4h}$)<br>$^4A_{2g} \rightarrow {}^4E_g$($D_{4h}$) |
| **17** (grayish-green) | 411<br>561<br>661 | 24,330<br>17,825<br>15,130 | $^4A_{2g} \rightarrow {}^4T_{1g}$<br>$^4A_{2g} \rightarrow B_{2g}$ ($D_{4h}$)<br>$^4A_{2g} \rightarrow {}^4E_g$($D_{4h}$) |

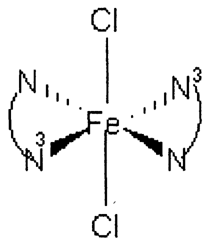

**6**

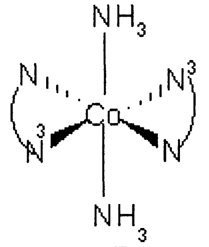

**7**

In the case of the copper complexes **13-15**, the electronic spectroscopy gave very few stereochemical information regarding the structure of the obtained compounds. Still, data of Table III, correlated with the measured magnetic moments and EPR spectra, allowed us to propose some hypothetical structures. Thus, the only Cu(II) complex for which EPR signals were evidenced (at room temperature, in powder) was **14**, for which an isotropic EPR spectrum with g = 2.0647 was obtained. We hypothesize that this compound contains a Cu(II) and a Cu(I) ion, and probably the same is true for the polymeric derivative **15**. The other obtained Cu(II) derivative, **13**, probably contains Cu(II) in trigonal bipyramidal geometry.[41] Since good crystals for X-ray crystallographic experiments could not be obtained (mainly due to the extremely low solubility of the obtained complexes in may solvents, excepting DMSO and DMF), all the structures proposed by us are tentative, but they are based on data previously obtained by our and Borras' group, by means of X-ray crystallography.[21-23]

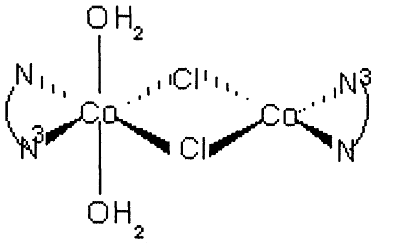

9

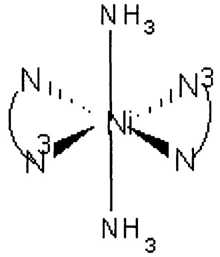

11

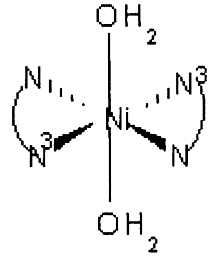

12

Table V: IR, thermogravimetric and conductimetric data for compounds **5-17** and the original ligands.

| Compound. | IR Spectra[a], $cm^{-1}$ | | | | | TG analysis[b] | Conductimetry[c] |
|---|---|---|---|---|---|---|---|
| | $\nu(SO_2)^s$ | $\nu(SO_2)^{as}$ | $\nu$(C=N) | $\nu$(CO) | $\nu$(M-X) | calc./found | $\Lambda_M$ ($\Omega^{-1}$ x $cm^2$x $mol^{-1}$) |
| **Htda** | 1170 | 1350 | 1610 | - | - | d | 0.9 |
| **3** | 1170 | 1350 | 1610 | 1720 | - | d | 0.8 |
| **5** | 1170 | 1360 | 1610 | 1720 | 445 | d | 279 |
| **6** | 1170 | 1340 | 1600 | 1720 | 315, 390 | d | 3 |
| **7** | 1150 | 1360 | 1600 | 1720 | 510 | 5.6/5.8[e] | 9 |
| **8** | 1150 | 1370 | 1600 | 1720 | 420, 530 | 5.1/5.0[f] | 11 |
| **9** | 1175 | 1350 | 1590 | 1720 | 395, 470 | 5.3/5.2[f] | 298 |
| **10** | 1170 | 1340 | 1600 | 1720 | 420, 505 | 3.2/3.2[f] | 2 |
| **11** | 1150 | 1360 | 1575 | 1720 | 530 | 5.6/5.8[e] | 6 |
| **12** | 1150 | 1355 | 1590 | 1720 | 435, 540 | 5.9/6.0[f] | 4 |
| **13** | 1150 | 1350 | 1600 | 1720 | 420, 520 | d | 2 |
| **14** | 1130 | 1360 | 1600 | 1695 | 310, 415 | d | 9 |
| **15** | 1145 | 1370 | 1595 | 1695 | 295, 430 | d | 3 |
| **16** | 1150 | 1350 | 1600 | - | 390 | d | 2 |
| **17** | 1140 | 1350 | 1600 | - | 450 | 21.7/21.4[g] | 114 |

[a] In KBr; [b] Weight loss between 70-190 °C; [c] 1 mM solution, in DMF, at 25°C; [d] No weight loss seen under 250 °C; [e] Corresponding to $2NH_3$, lost at 135-150 °C; [f] Corresponding to $2H_2O$ (coordinated), lost at 160-180 °C; [g] Corresponding to eight lattice water molecules lost at 70-110°C.

Both complexes containing the sulfonamide-deprotonated species of sulfonamides **3** (L), as well as complexes in which the free sulfonamide itself HL or its hydrolysis product, 5-amino-1,3,4-thiadiazole-2-sulfonamide (Htda) act as ligands, have been prepared in the present study (Table I). By comparing the IR spectra of the complexes and the corresponding ligands (Table V), the following observations should be made: (i) the shift of the two sulfonamido vibrations (both the symmetric as well as the the assymetric one), towards lower or higher wavenumbers in the spectra of the complexes, as compared to the spectra of the corresponding ligand, as already documented previously for similar complexes.[1,21-27] This is a direct indication that the (deprotonated) sulfonamido moieties of the ligands interacts with the metal ions in the newly prepared coordination compounds; (ii) the amide vibrations (the most intense such bands at 1720 $cm^{-1}$) of ligand **3** appear unchanged in the IR spectra of complexes **5-13**, suggesting that these moieties do not participate in coordination of the metal ions. Still, this band is shifted at 1695 $cm^{-1}$ in the spectra of the two copper complexes **14** and **15**, probably due to participation of this moiety in coordination of the metal ions ; (iii) the C=N stretching vibration in the spectra of the prepared complexes is shifted with 5-20 $cm^{-1}$ towards lower wavenumbers, as compared to the same vibration in the spectra of sulfonamides **3** and Htda, indicating that one of the endocyclic nitrogens of the thiadiazolic ring (presumably N-3) acts as donor atom, as already documented by X-ray crystallographic and spectroscopic determinations on complexes of other sulfonamides (such as **1-3**) with divalent metal ions (Table V);[1,21-27] (iv) changes in the region 3100-3160 $cm^{-1}$, as the bands present in the spectra of the original sulfonamides are present in the spectra of the prepared complexes too, but they are not well resolved, and have a smaller intensity (data not shown). This is probably due to deprotonation of the $SO_2NH_2$ moiety and participation in the binding of cations; (v) the presence of M-O, M-N or M-Cl vibrations in the region between 295-550 $cm^{-1}$.

Thermogravimetric analysis showed the presence of coordinated water/ammonia molecules in some of the prepared complexes, which were lost in a single step, between 135-180 °C, and of lattice water in the case of one complex, **17**. All these compounds behaved as non-electrolytes in DMF as solvent, except for **5** and **9** (2:1, and 1:2 electrolytes, respectively) and **17** (1:1 electrolyte) (Table V).

By correlating all the spectroscopic, magnetic, TG and conductimetric data, one can conclude that ligand L (the conjugate base of sulfonamide **3**) generally acts as a bidentate ligand in the complexes prepared here, interacting with the metal ions by means of the sulfonamido and endocyclic N-3 nitrogen atoms. An exception is the V(IV) derivative in which we consider L to act monodentately, by means of the sulfonamidic nitrogen. When HL interacts with metal ions, the donor system is probably similar to that of L, but the obtained complexes are generally much less stable (data not shown). 5-Amino-1,3,4-thiadiazole-2-sulfonamide Htda also acts as a bidentate ligand, by the same donor system as sulfonamide **3**, similarly with the situation described before,[1] when the Mg(II); Be(II); Zn(II); Cd(II), Hg(II) and Pb(II) complexes of this sulfonamide have been reported.

Table VI. CA inhibition data with the standard inhibitors **1-3**, and the metal complexes **5-17** against isozymes CA I, II and IV.

| No | Inhibitor | hCA I[a] | $K_I$ (nM) hCA II[a] | bCA IV[b] |
|---|---|---|---|---|
| **1** | Acetazolamide | 900 | 12 | 220 |
| **2** | Benzolamide | 780 | 14 | 240 |
| **3** | LH | 640 | 5.0 | 24 |
| | Htda | 1550 | 230 | 780 |
| **5** | | 230 | 4.1 | 19 |
| **6** | | 255 | 4.4 | 16 |
| **7** | | 180 | 3.6 | 12 |
| **8** | | 360 | 4.2 | 21 |
| **9** | | 200 | 3.9 | 15 |
| **10** | | 175 | 3.5 | 13 |
| **11** | | 220 | 3.7 | 14 |
| **12** | | 205 | 3.5 | 11 |
| **13** | | 190 | 3.2 | 9 |
| **14** | | 85 | 2.5 | 8 |
| **15** | | 155 | 3.9 | 12 |
| **16** | | 740 | 87 | 540 |
| **17** | | 680 | 79 | 490 |

[a] Human (cloned) isozymes; [b] From bovine muscle; [c] From bovine lung microsomes.

The complexes **5-17** together with the standard CA inhibitors **1-3** were assayed for inhibition against four isozymes, hCA I, hCA II, and bCA IV (Table VI). As seen from the above data, the chloracetamido derivative **3** is more inhibitory than acetazolamide, whereas the unacylated compound Htda is less inhibitory than the above sulfonamides. The metal complexes **5-17** are much more inhibitory than the sulfonamides from which they derive **3**, and Htda, and than all other simple sulfonamides assayed. They behave similarly to the metal complexes of acetazolamide, methazolamide or dorzolamide previously reported by this group, which were all more inhibitory than the parent sulfonamide from which were prepared. [1,22-27] Particularly strong inhibition was observed for the Co(II); Ni(II) and Cu(II) complexes, especially against CA II and CA IV, the isozymes critical for aqueous humor formation.

The above differences of affinity for the different isozymes of the new inhibitors reported in this paper, and the fact that the X-ray crystallographic structure of hCA IV has been recently reported[42] and became available in the Brookhaven Protein Database (in September 1997; file code 1ZNC), prompted us to compare the tridimensional structure and active site architecture for the three CA isozymes (CA I, II and IV) used in the present work (human and bovine CA IV possess

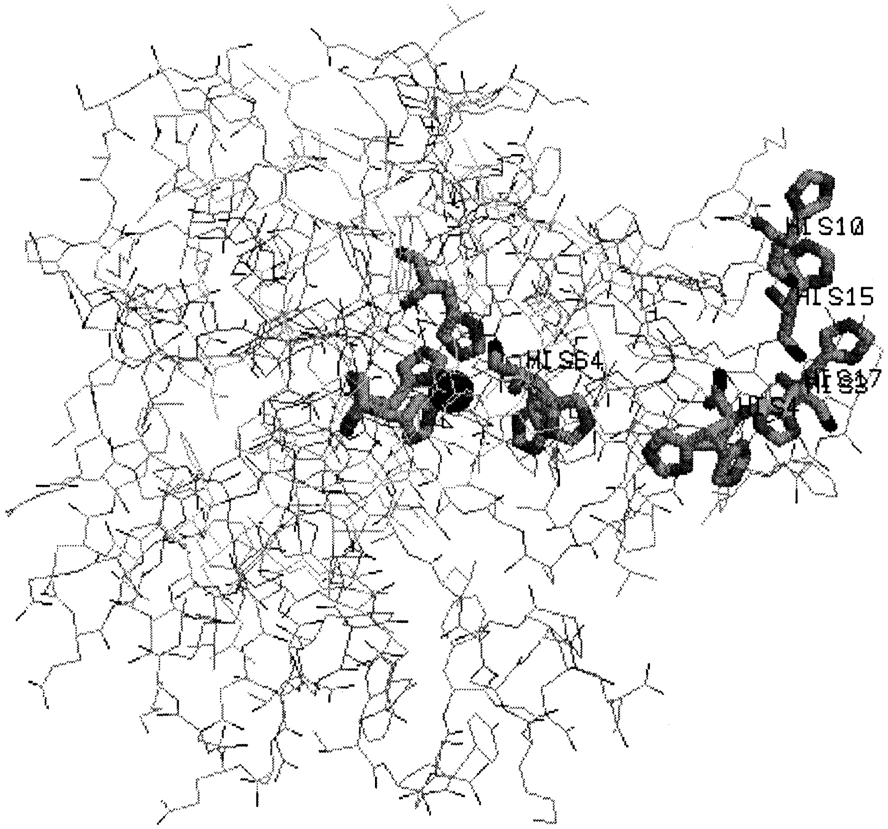

Fig. 1: hCA II with the catalytically critical Zn(II) ion (central sphere) and its three histidine ligands (His 94, His 96 and His 119), as well as the histidine cluster evidenced. The figure has been generated by using the programme RasMol for Windows 2.6 with a Texas Instruments 4000 M PC, from the X-ray crystallographic coordinates of Hakansson et al.,[46] available from Brookhaven Protein Database (file code 2CBA).

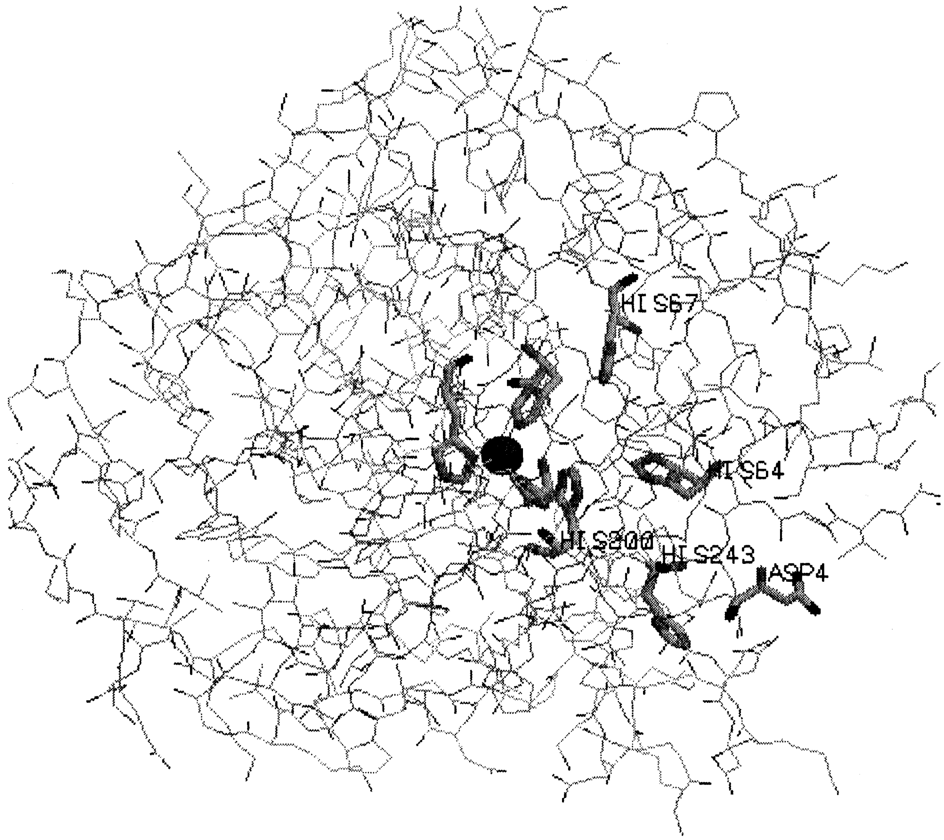

Fig. 2: hCA I with the catalytically critical Zn(II) ion (central sphere) and its three histidine ligands (His 94, His 96 and His 119), as well as the active site histidine residues and Asp 4 evidenced. The figure has been generated by using the programme RasMol for Windows 2.6 with a Texas Instruments 4000 M PC, from the X-ray crystallographic coordinates of Chakravarti and Kannan[45] available from Brookhaven Protein Database (file code 1HUH).

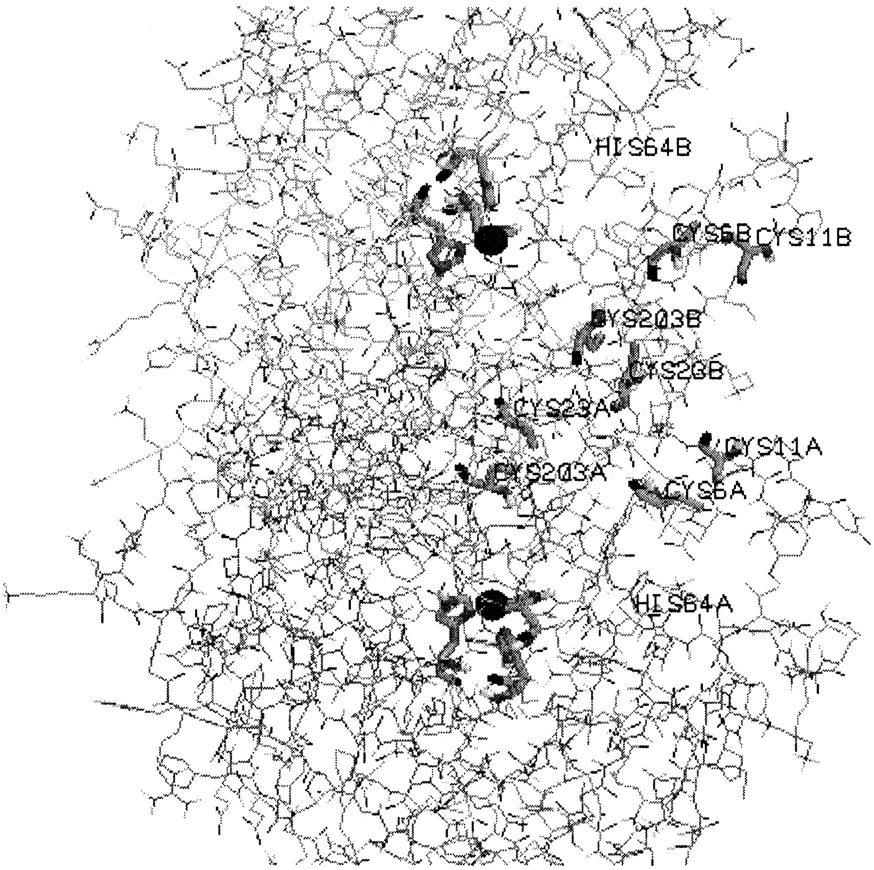

Fig. 3: hCA IV with the catalytically critical Zn(II) ion (central spheres) and their three histidine ligands (His 94, His 96 and His 119), as well as the active site residues His 64; Cys 6; Cys 11G; Cys 23 and Cys 203 evidenced. Two crystallographically non-equivalent protein molecules are present in the cell unit of hCA IV.[42]

very similar kinetic and inhibition susceptibility properties[43]) in order to explain their affinity for the sulfonamide inhibitors reported by us here. Thus, Figs 1-3 show some active site residues known to be important for catalysis in isozymes I, II and IV[43-46] (the files 1HUH for hCA I and 2CBA for hCA II have been used, also available from Brookhaven Protein Database).[43-46]

It has previously been proposed by us[44] that the high catalytic efficiency of the most active isozyme, hCA II, is due to a unique feature of its active site: the presence of a histidine cluster, consisting of the residues: His 64 - two conformations; His 4 - two conformations, His 3, His 10, His 15, His 17 (Fig. 1).[44] This cluster extends from the interior of the active site (His 64) to its entrance (His 4 and His 3) till the surface of the protein (in the proximity of the active site entrance) and it probably constitutes a very appropriate "channel" for efficiently transferring protons from the active site to the reaction medium, but also for the binding of amphipatic compounds, such as the sulfonamide inhibitors and their metal complexes.[44,47] As seen from Fig. 2, in the low activity isozyme hCA I (which also binds sulfonamide/metal complexes of sulfonamide inhibitors with a 10-100 times lower affinity), such a cluster does not exist.[44] Moreover, the pathways for the proton transfer are somehow bifurcated and divergent as the four histidines present within the active site, ie, His 64, 67, 200 and 243 (excepting, of course, for the three Zn(II) ligands, which in all isozymes are His 94, 96 and 119) are placed at bifurcating positions. As seen from Fig. 2, these four histidines in hCA I (His 64, His 67, His 200 and His 243) are rather buried in the active site so that, probably, the proton transfer cannot be as efficient as the one assisted by the histidine cluster present in HCA II, whereas inhibitors are probably unable to interact with them, as documented by crystallographic studies of adducts of hCA I with sulfonamides and anion inhibitors.[45] In this context it is also enlightening that histamine, the first activator of hCA II for which the X-ray crystal structure has recently been reported by this group,[44] which is able to enhance the catalytic efficiency of the enzyme, does really bind in the region of the active site of hCA II containing the histidine cluster shown in Fig. 1. In this context, we suggest a new hypothesis for explaining also differences of affinity of the discussed CA isozymes for the sulfonamide inhibitors and their metal complexes, based on the interaction of inhibitors with the structural elements of the active site mentioned above. Indeed, for example in hCA II the amino acid residue in position 4 is His, whereas in hCA I it is an Asp. This residue, situated just at the entrance within the active site, is a constituent of the histidine cluster of isozyme II. The negative charge of the carboxylate group of the Asp residue in hCA I probably influences its interaction with positive charges (such as the metal ions), present in the complex inhibitors of the type described by us here. In fact many such compounds have a higher affinity for hCA I as compared to the parent, uncharged sulfonamides from which they were obtained, presumably due to a supplementary interaction between this negative charge and the cationic moiety of the inhibitor molecule.

In the case of hCA IV (Fig. 3) only one histidine residue is present within the active site, His 64, which as in hCA II, plays a critical role in catalysis, as proton shuttle residue between the active site and the environment. But the most characteristic feature of the active site of this isozyme is related to the presence of four cysteine residues, which form two disulfide bonds, situated at the entrance within the active site cavity (Cys 6 - Cys 11G, and Cys 23 - Cys 203, respectively). These residues occupy practically the same region of the active site as the histidine cluster in hCA II, and we consider this as the most relevant aspect explaining the difference in affinity for sulfonamide inhibitors of the two isozymes. The hypothesis of Stams et al.[42] that it is the residue 131 (a valine in hCA IV and a phenylalanine in hCA II) which is responsible for the above mentioned differences, seems to us very unrealistic, since this residue is situated relatively far away from the active site, and could account for such a difference in affinity only for inhibitors with an extremely long and flexible molecule.

In conclusion, in this paper we present novel types of very potent CA inhibitors from the class of the metal complexes of heterocyclic sulfonamides, together with a new hypothesis to explain the different affinity of such inhibitors for the physiologically relevant isozymes CA I, II and IV.

### References

1 Preceding part of this series: C.T. Supuran, A. Scozzafava, A. Jitianu, *Metal Based Drugs*, **1997**, *4*, in press.

2. a) R.O. Roblin, J.W. Clapp, *J.Am.Chem.Soc.*, **1950**, *72*, 4890-4892; b) W.H. Miller, A.M. Dessert, R.O. Roblin, *J.Am.Chem.Soc.*, **1950**, *72*, 4893-4896; c) J.R. Vaughan, J.A. Eichler, G.W. Anderson, *J.Org.Chem.*, **1956**, *21*, 700-701; d) R.W. Young, K.H. Wood, J.R. Vaughan, G.W. Anderson, *J.Am.Chem.Soc.*, **1956**, *78*, 4649-4654.

3. a) C.T. Supuran, *Roum.Chem.Quart.Rev.*, **1993**, *1*, 77-116; b) C.T. Supuran, "Carbonic anhydrase inhibitors", in "Carbonic Anhydrase and Modulation of Physiologic and Pathologic Processes in the Organism", I. Puscas Ed., Helicon, Timisoara **1994**, pp. 29-111.

4. a) T.H. Maren, *Pharmacol.Rev.*, **1967**, *47*, 595-782; b) C.T. Supuran, C.W. Conroy, T.H. Maren, *Eur. J. Med. Chem., 31,* **1996**, 843-846.

5. T.H. Maren, *Annu.Rev.Pharmacol.Toxicol.*, **1976**, *16*, 309-327.

6. K.H. Bayer, J.E. Baer, *Pharmacol. Rev.*, **1961**, *13*, 517-562.

7. a) T.H. Maren, *J. Glaucoma*, **1995**, *4*, 49-62; b) S. Lindskog, P.J. Wistrand. " Inhibition of carbonic anhydrase ", in "Design of Enzyme Inhibitors as Drugs", M.J. Sandler, H.J. Smith Eds., Oxford Univ. Press, Oxford, **1987**, pp. 698-723; c) T.H. Maren, C.W. Conroy, *J.Biol.Chem.*, **1993**, 268, 26233-26238.

8. M.F. Sugrue *J. Ocul. Pharmacol.* ***Ther.,*** **1996**, *12*, 363-376.

9. J.D. Bartlett, S.D. Jaanus "Carbonic anhydrase inhibitors". In “Clinical Ocular Pharmacology”, Second Edition, Butterworths Publishers, Boston, 1989, pp. 254-263.

10. W.G. Reiss, K.S. Oles *Ann. Pharmacother.,* **1996**, *30*, 514-519.

11. I. Puscas, C.T. Supuran, "Farmacologia clinica da ulcera peptica" in "Aparelho Digestivo", J. Coelho Ed., MEDSI, Rio de Janeiro, **1996**, pp. 1704-1734.

12. a) M. Stoll, G.F. Hamann, V. Jost, U.A. Bompotti, R. Fitridge, K. Schimrigk, *J. Neuroimaging*, **1996**, *6*, 144-149; b) R.L. Levine, P.A. Tursky, W.D. Turnipseed, T. Grist, *J. Neuroimaging*, **1996**, *6*, 126-130.

13. a) T.H. Maren, L. Jankowska, G.F. Edelhauser, G. Sanyal, *Exp.Eye Res.*, **1983**, *36*, 457-480; b) J.P. Tinker, R. Coulson, I.M. Weiner, *J. Pharmacol. Exp. Ther.*, **1981**, *218*, 600-607; c) M.S. Lucci, J.P. Tinker, I.M. Weiner, T.D. duBose, *Am. J. Physiol.*, **1983**, *245*, F443-F449.

14. a) C.T. Supuran, G. Manole, A. Dinculescu, A. Schiketanz, M.D. Gheorghiu, I. Puscas, A.T. Balaban, *J. Pharm. Sci.*, **1992**, *81*, 716-719; b) C.T. Supuran, A.T. Balaban, M.D. Gheorghiu, A. Schiketanz, A. Dinculescu, I. Puscas, *Rev. Roum. Chim.*, **1990**, *35*, 399-405.

15. a) C.T. Supuran, B.W. Clare, *Eur. J. Med. Chem.,* **1995**, *30,* 687-696; b) C.T. Supuran, A. Popescu, M. Ilisiu, M.D. Banciu, *Eur. J. Med. Chem., 31,* **1996**, 439-448; c) A. Scozzafava, C.T. Supuran, *Metal Based Drugs,* **1997**, *4,* 19-26; d) C.T. Supuran, M.D. Banciu, A. Popescu, *Rev.Roum.Chim.*, **1992**, *37*, 289-297.
16. a) G. Mincione, A. Scozzafava, C.T. Supuran, *Metal Based Drugs,* **1997**, *4,* 27-34; b) C.T. Supuran, A. Scozzafava, *J. Enzyme Inhib.,* **1997**, *12*, 37-51; c) C.T. Supuran, F. Briganti, A. Scozzafava, *J. Enzyme Inhib.,* **1997**, *12*, 175-190.
17. T.H. Maren, "Benzolamide", in "Orphan Drugs", F.E. Karch Ed., M. Dekker, New York, 1982, pp. 89-115.
18. E.R. Swenson, T.H. Maren, *Respir. Physiol.,* **1978**, *35*, 129-159.
19. W.F. Brechue, T.H. Maren, *Invest. Ophthalmol. Vis. Sci.,* **1993**, *34*, 2581-2587.
20. a) A. Bayer, F. Ferrari, T.H. Maren, C. Erb, *J. Fr. Ophtalmol.,* **1996**, *19*, 357-362; b) T.H.Scholz, J.M. Sondey, W.C. Randall, H. Schwam, W.J. Thompson, P.J. Mallorga, M.F. Sugrue, S.L. Graham, *J.Med.Chem.*, **1993**, *36*, 2134-2141.
21. a) S. Ferrer, A. Jimenez, J. Borras, *Inorg. Chim. Acta*, **1987**, *129*, 103-106; b) S. Ferrer, , J. Borras, C. Mirratvilles, A. Fuertes, *Inorg. Chem.*, **1989**, *28*, 160-163.
22. a) C.T. Supuran, M. Andruh, I. Puscas, *Rev.Roum.Chim.*, **1990**, *35*, 393-398; b) C.T. Supuran, R. Stefan, G. Manole, I. Puscas, M. Andruh, *Rev.Roum.Chim.*, **1991**, *36*, 1175-1179; c) C.T. Supuran, G. Manole, M. Andruh, *J.Inorg.Biochem.,* **1993**, *49,* 97-103; d) C.T. Supuran, *Rev.Roum.Chim.*, **1992**, *37*, 849-855; e) C.T. Supuran, M. Andruh, *Rev.Roum.Chim.*, **1994**, *39*, 1229-1234.
23. a) G. Alzuet, S. Ferrer, J. Borras, C.T. Supuran, *Roum.Chem.Quart.Rev.*, **1994**, *2*, 283-300; b) S.L. Sumalan, J. Casanova, G. Alzuet, J. Borras, A. Castiñeiras, C.T. Supuran, *J.Inorg.Biochem.*, **1996**, 62, 31-39; c) C.T. Supuran, G.L. Almajan, *Main Group Met. Chem.*, **1995**, *18*, 347-351.
24. a) C.T. Supuran, *Metal Based Drugs*, **1995**, *2*, 327-330; b) C.T. Supuran, *Metal Based Drugs*, **1995**, *2*, 331-336; c) J. Borras, T. Cristea, C.T. Supuran, *Main Group Met. Chem.*, **1996**, *19*, 339-346; d) J. Borras, J. Casanova, T. Cristea, A. Gheorghe, A. Scozzafava, C.T. Supuran, V. Tudor, *Metal Based Drugs*, **1996**, *3*, 143-148; e) C.T. Supuran, *Rev.Roum.Chim.*, **1993**, *38*, 229-236.
25. a) A. Jitianu, M.A. Ilies, A. Scozzafava, C.T. Supuran, *Main Group Met. Chem.*, **1997**, 20, 147-153; b) C.T. Supuran, *Main Group Met. Chem.*, **1996**, *19*, 347-354.
26. C.T. Supuran, F. Mincione, A. Scozzafava, F. Briganti, G. Mincione, M.A. Ilies, *Eur. J. Med. Chem.,* **1998**, *33,* in press.
27. C.T. Supuran, A. Scozzafava, I. Saramet, M.D. Banciu, *J. Enzyme Inhib.,* **1998**, *13*, in press.
28. D. Hewett-Emmett, R.E. Tashian, *Mol.Phylogenet.Evol.*, **1996**, *5*, 50-77.
29. R.E. Tashian, D. Hewett-Emmett, P.J. Venta, in "Carbonic anhydrase – From Biochemistry and Genetics to Physiology and Clinical Medicine", F. Botrè, G. Gros, B.T. Storey, Eds., Weinheim, 1991, pp. 151-161.
30. N. Carter, in "Carbonic anhydrase – From Biochemistry and Genetics to Physiology and Clinical Medicine", F. Botrè, G. Gros, B.T. Storey, Eds., Weinheim, 1991, pp. 168-177.
31. R.S. Drago, in "Physical Methods in Chemistry", W.B. Saunders & Co., London, **1977**, p. 411.
32.a) C. Forsman, G. Behravan, A. Osterman, B.H. Jonsson, *Acta Chem. Scand.*, **1988**, *B42*, 314-318; b) G. Behravan, P. Jonasson, B.H. Jonsson, S. Lindskog, *Eur.J.Biochem.*, **1991**, *198*, 589-592.
33. R.G. Khalifah, D.J. Strader, S.H. Bryant, S.M. Gibson, *Biochemistry*, **1977**, *16*, 2241-2247.
34. a) P.O. Nyman, S. Lindskog, *Biochim.Biophys.Acta*, **1964**, *85*, 141-151; b) L.E. Henderson, D. Henriksson, P.O. Nyman, *J.Biol.Chem.*, **1976**, *251*, 5457-5463.
35. T.H. Maren, G.C. Wynns, P.J. Wistrand, *Mol. Pharmacol.,* **1993**, *44*, 901-906
36. Y. Pocker, J.T. Stone, *Biochemistry*, **1967**, *6*, 668-679.
37. C.J. Balhausen, H. Gray, Inorg. Chem., **1962**, *1*, 111-115.
38. A.B.P. Lever, "Inorganic Electronic Spectroscopy", 2nd Edition, Elsevier, Amsterdam, **1984**, pp. 135-138.
39. L. Sacconi, F. Mani, A. Bencini, "Nickel", in "Comprehensive Coordination Chemistry", G: Wilkinson, R. Gillard, J. McCleverty Eds., Pergamon Press, Oxford, **1987**, Vol. 5, pp. 1-347.
40. L. Banci, A. Bencini, C. Benelli, D. Gatteschi, C. Zanchini, *Struct. Bonding*, **1982**, *52*, 37-79.
41. B.J. Hathaway, "Copper", in "Comprehensive Coordination Chemistry", Vol. 5, G. Wilkinson, R.D. Gillard, J. Cleverty Eds., Pergamon, New York, 1987, pp. 533-546.
42 T. Stams, S.Kk Nair, T. Okuyama, A. Waheed, W.S. Sly, D.Ww Christianson, *Proc. Natl. Acad. Sci. USA,* **1996**, *93*, 13589-13594.

43 S. Tamai, A. Waheed, L.B. Cody, W.S. Sly, *Proc. Natl. Acad. Sci USA,* **1996**, *93*, 13647-13652.
44 F. Briganti, S. Mangani, P. Orioli, A. Scozzafava, G. Vernaglione, C.T. Supuran, *Biochemistry,* **1997**, *36*, 10384-10392.
45 S. Chakravarti, K.K. Kannan, *J. Mol. Biol.,* **1994**, *243*, 298-309.
46 K. Håkansson, M. Carlsson, L.A. Svensson, A. Liljas, *J. Mol. Biol.,* **1992**, *227*, 1192-1204.
47 D. Elbaum, S.K. Nair, M.W. Patchan, R.B. Thompson, D.W. Christianson, *J. Am. Chem. Soc.,* **1996**, *118*, 8381-8387.

**Received: February 9, 1998 - Accepted: March 6, 1998 - Received in revised camera-ready format: March 9, 1998**

